# Dual effects of 9-*cis* retinoic acid on ACTH-dependent hyperplastic adrenal tissues

**DOI:** 10.1038/s41598-021-93672-0

**Published:** 2021-07-12

**Authors:** Francesca Pecori Giraldi, Antonella Sesta, Laura Tapella, Maria Francesca Cassarino, Luigi Castelli

**Affiliations:** 1 Department of Clinical Sciences and Community Health, University of Milan, 20122 Milan, Italy; 2 Neuroendocrinology Research Laboratory, Istituto Auxologico Italiano IRCCS, Via Zucchi 18, 20095 Cusano Milanino, MI Italy; 3 Ospedale San Carlo, Reparto di Chirurgia, 20037 Paderno Dugnano, MI Italy

**Keywords:** Adrenal gland diseases, Translational research, Endocrinology

## Abstract

Retinoids play a pivotal role in adrenal development and differentiation. Recent clinical trials revealed therapeutic potential of both all-*trans* and 9-*cis* retinoic acid in patients with cortisol excess due to a pituitary ACTH-secreting adenoma and indicated that retinoids might act also on the adrenal. Aim of the present study was to evaluate the effect of 9-*cis* retinoic acid on adrenals from patients with ACTH-dependent Cushing’s syndrome. Adrenal specimens from six patients with Cushing’s disease were incubated with 10 nM–1 µM 9-*cis* retinoic acid with and without 10 nM ACTH. Cortisol secretion was measured by immunoassay and expression of genes involved in steroidogenesis as well as retinoic acid action were evaluated by real-time RT-PCR. Incubation with 10–100 nM 9-*cis* retinoic acid increased spontaneous cortisol secretion and expression of *STAR* and *CYP17A*. On the other hand, in wells treated with ACTH, 9-*cis* retinoic acid markedly diminished ACTH receptor upregulation and no stimulatory effect on cortisol secretion or steroidogenic enzyme synthesis was observed. ACTH itself increased ligand-induced retinoic acid receptor expression, possibly enhancing sensitivity to retinoic acid. Our findings indicate that the effect of 9-*cis* retinoic acid in presence of ACTH is distinct from unchallenged wells and support the hypothesis of a direct adrenal action in patients with Cushing’s disease.

## Introduction

Retinoids are critical for embryonal organogenesis, in particular neural crest and mesoderm-derived organs including the adrenal gland. Several lines of evidence support the role of retinoids in early stage adrenal differentiation^1^ and zona fasciculata organization^2^. In adult life, retinoids are known to exert an antiproliferative effect in a variety of cells, including skin, breast and neuronal cells^3^, and currently play a role in treatment of acute promyelocytic leukemia^4^ and possibly other tumors, such as, neuroblastoma, breast cancer, melanoma, ^3^. Retinoic acid has also been tested in adrenal cancer and shown to modulate corticosteroid secretion and cell proliferation^5,6^.

Most recently, retinoids have been proposed for the treatment of Cushing's disease, a severe endocrine disorder caused by an excess cortisol secretion due to a pituitary corticotropin (ACTH)-secreting tumor^7^. In vitro studies revealed that all-*trans* retinoic acid as well as the 9-*cis* derivate inhibit proliferation in a murine corticotrope tumor cell line and blunt ACTH secretion in human corticotrope adenomas^5,8–10^. Our pilot study in patients with Cushing's disease revealed that all*-trans* retinoic acid (tretinoin) administration is beneficial in these patients^11^ and a subsequent study with the 13-*cis* isomer isotretinoin confirmed these promising results^12^. In detail, administration of tretinoin or isotretinoin reduced markers of cortisol excess in all patients and normalization of urinary free cortisol levels was achieved in approximately 30% of patients^11,12^. Amelioration of clinical parameters of Cushing’s disease, e.g., blood pressure, weight, glucose control, hirsutism, was also observed during tretinoin or isotretinoin treatment^11,12^.

Interestingly, the decrease in cortisol secretion during either retinoid was more pronounced that the change in ACTH levels. While this phenomenon is not uncommon in patients with Cushing’s disease treated with pituitary-acting drugs^13,14^, it could be due to a direct action on adrenal glands. In our first study on normal adrenal cortex tissue, we observed a dual effect of 9-*cis* retinoic acid: stimulation of cortisol secretion and *STAR* expression by roughly 1.5-fold on one side and halving of ACTH receptor synthesis on the other^15^. We thus hypothesized that the efficacy of retinoids in Cushing’s disease could also be due to a direct effect on the adrenal gland and decided to test this hypothesis in hyperplastic adrenal tissue from these patients.

Aim of the present study was to assess the effects of 9-*cis* retinoic acid on cortisol secretion and on genes involved in steroidogenesis and retinoid action in adrenal glands from patients with Cushing’s disease. In detail, we evaluated 17hydroxylase, StAR (steroidogenic acute regulatory protein), hormone-sensitive lipase E (LIPE), the ACTH receptor (MC2R), as well as known retinoid target genes, such as retinoic acid receptors alpha and beta, liver X receptor (LXR), peroxisome proliferator activated receptor delta (PPARD), chicken ovoalbumin upstream promoter transcription factor 1 (COUP-TF1), sterol regulatory element binding transcription factor 1 (SREBP1), mitochondrial dehydrogenases mND1 and mND6, and genes involved in both pathways, e.g., dosage-sensitive sex-reversal adrenal hypoplasia critical region in the X chromosome (DAX-1) and steroidogenic factor 1 (SF-1).

## Results

**Figure 1:**
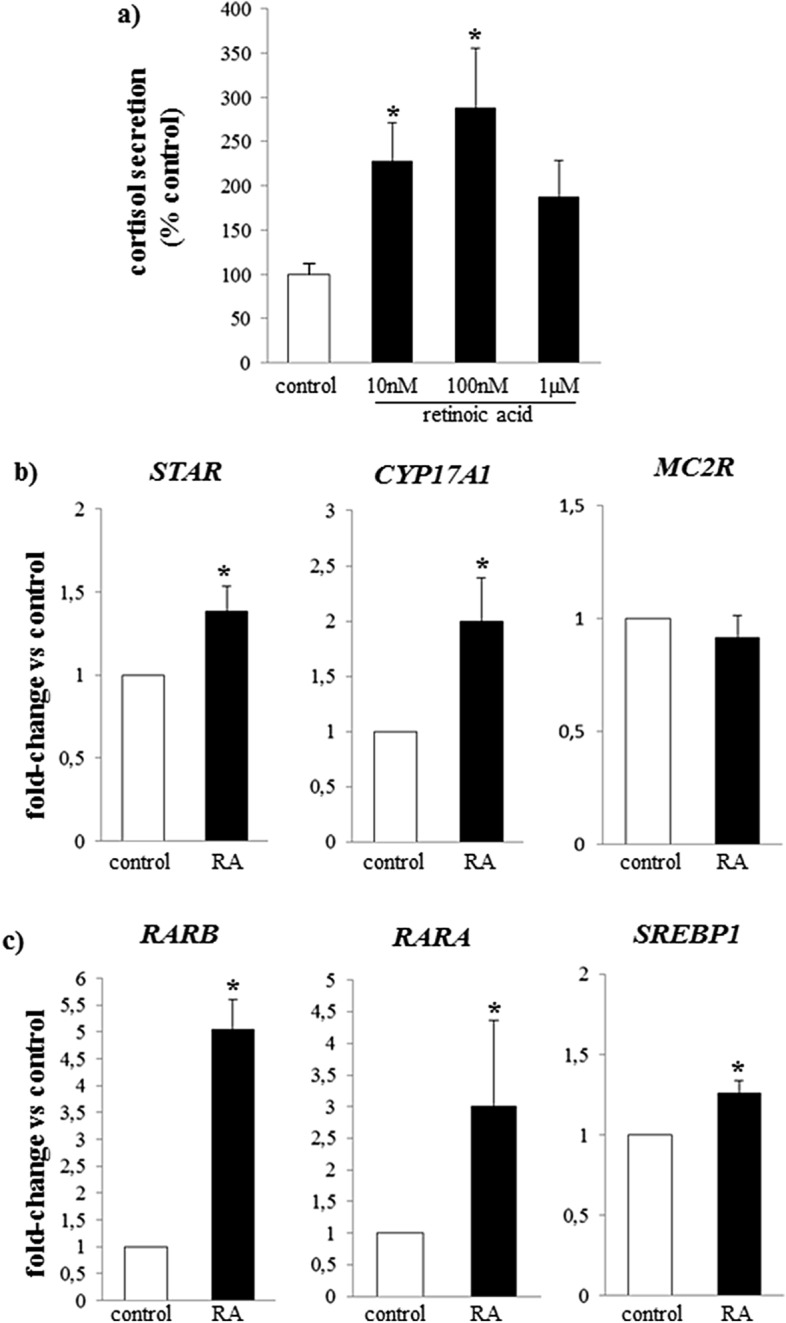
Effect of 9-*cis* retinoic acid on baseline cortisol secretion and gene expression in adrenal cultures from patients with Cushing’s disease. (**a**) Mean cortisol secretion after 24 h incubation with 10 nM–1 µM 9-*cis* retinoic acid; (**b**) expression of *STAR*, *CYP17A1* and *MC2R* after 24 h incubation with 10 nM 9-*cis* retinoic acid (RA); (**c**) expression of *RARA*, *RARB* and *SREBP1* after 24 h incubation with 10 nM 9-*cis* retinoic acid (RA). Data is expressed relative to untreated, control wells: equal to 100% for cortisol secretion and equal to 1 for gene expression. White bar: control, black bars: 9-*cis* retinoic acid (RA). *p < 0.05 vs control.

9-*cis* Retinoic acid increased cortisol secretion in adrenal primary cultures from patients with ACTH-dependent Cushing’s syndrome. Cortisol concentrations on average doubled with respect to control wells for both 10 nM and 100 nM 9-*cis* retinoic acid (Fig. 1a, both p < 0.05); a lesser increase in cortisol was observed with 1 µM 9-*cis* retinoic acid (Fig. 1a). 9-*cis* Retinoic acid also increased expression of *StAR* and *CYP17A* (Fig. 1b), and decreased *NR0B1* (DAX1, Table 1) compared to untreated wells.

**Table 1:** Gene expression in adrenal cultures from patients with Cushing’s disease.

	10 nM ACTH	10 nM retinoic acid#	retinoic acid + ACTH#	retinoic acid + ACTH^
**Genes involved in adrenal steroidogenesis**				
*CYP17A1*	3.38 ± 0.763*	2.00 ± 0.393*	4.56 ± 1.204	1.48 ± 0.198
*STAR*	2.06 ± 0.306*	1.38 ± 0.149*	2.30 ± 0.277	1.38 ± 0.440
*NR5A1 (SF-1)*	1.26 ± 0.215	0.96 ± 0.076	0.96 ± 0.160	0.89 ± 0.172
*LIPE*	3.12 ± 0.596*	1.26 ± 0.220	2.25 ± 0.949	0.85 ± 0.091
*NR0B1 (DAX-1)*	1.00 ± 0.210	0.83 ± 0.131*	0.82 ± 0.086	1.00 ± 0.089
*MC2R*	4.42 ± 1.034*	0.92 ± 0.095	2.62 ± 0.58§	0.58 ± 0.037§
**Genes involved in retinoic acid action**				
*RARA*	1.44 ± 0.610	3.02 ± 1.352*	2.25 ± 0.949§	1.36 ± 0.152§
*RARB*	2.06 ± 0.195*	5.09 ± 0.540*	5.07 ± 0.580§	2.67 ± 0.258§
*NR2F1 (COUP-TF1)*	1.10 ± 0.170	0.83 ± 0.150	0.90 ± 0.155	0.82 ± 0.162
*PPARD*	1.06 ± 0.271	0.95 ± 0.096	1.06 ± 0.081	1.02 ± 0.124
*NR1H3 (LXRalfa)*	1.02 ± 0.174	1.32 ± 0.260	1.32 ± 0.174	1.22 ± 0.213
*SREBP1*	1.12 ± 0.235	1.26 ± 0.076*	0.93 ± 0.225	0.93 ± 0.192
*mt-ND-1*	1.50 ± 0.410	1.33 ± 0.243	1.66 ± 0.292	1.13 ± 0.079
*mt-ND-6*	0.80 ± 0.208	1.03 ± 0.127	0.87 ± 0.233	0.92 ± 0.107

^#^fold change relative to control wells; ^fold change relative to ACTH-stimulated wells; *p < 0.05 vs untreated wells; §p < 0.05 vs ACTH-treated wells. Data is expressed as mean ± S.E.M.

As regards the expression of genes related to the retinoic acid pathway, 9-*cis* retinoic acid increased the expression of both retinoic acid receptor alpha and beta and the transcription factor *SREBP1* (Fig. 1c). No significant changes during 9-*cis* retinoic acid treatment were observed for other factors involved in steroidogenesis, e.g., MC2R, SF-1, LIPE or in retinoic acid action, e.g., liver X receptor, PPARD, COUP-TF1 and the mitochondrial dehydrogenases*,* i.e., *mt-ND1, mt-ND6*, (Table 1), compared to control samples.

As expected, incubation with ACTH increased cortisol secretion (335.79 ± 134.64% control, p < 0.05 *vs* unchallenged wells) and induced the expression of *CYP17A1*, *LIPE* , *MC2R* and *StAR*. Interestingly, ACTH also induced *RARB* gene expression; no other retinoid acid-related gene was modified during ACTH incubation (Table 1).

**Figure 2:**
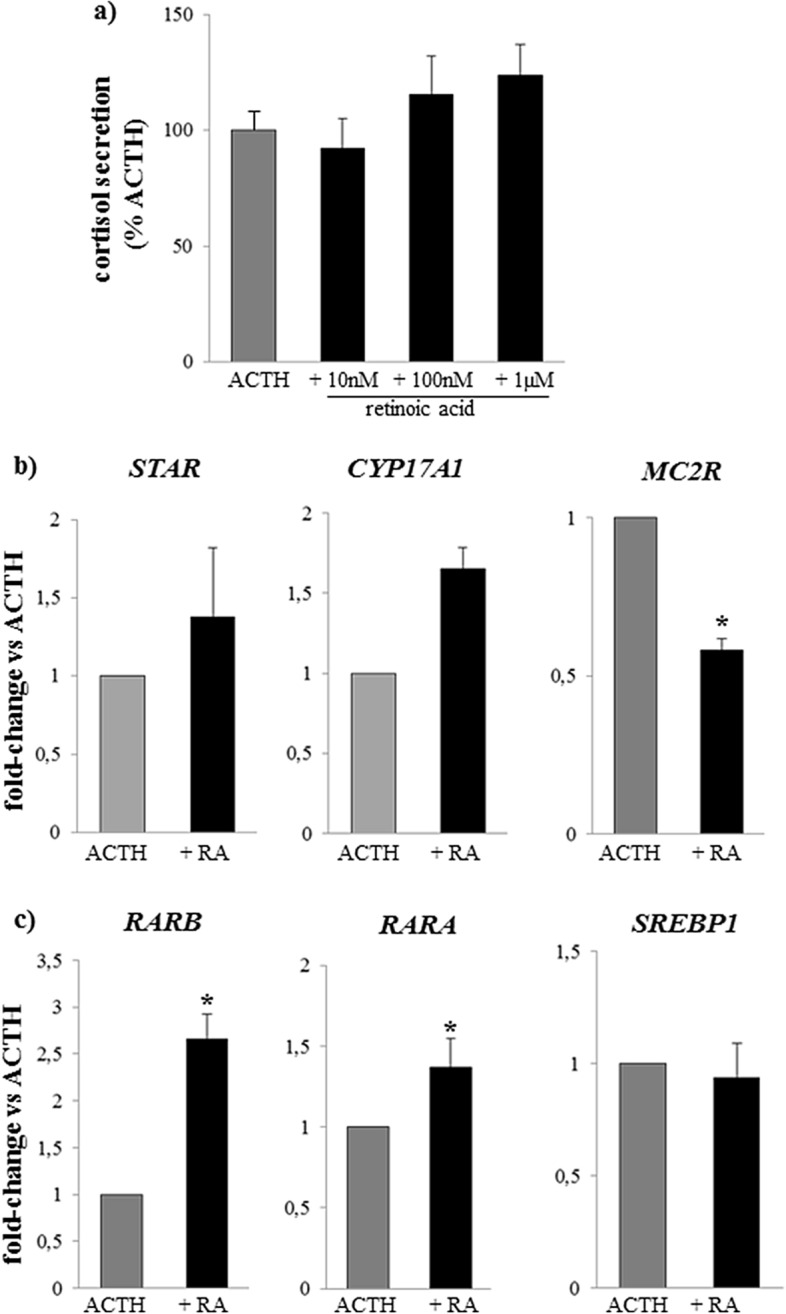
Effect of 9-*cis* retinoic acid on ACTH-stimulated cortisol secretion and gene expression in adrenal cultures from patients with Cushing’s disease. (**a**) mean ACTH-stimulated cortisol secretion after 24 h incubation with 10 nM–1 µM 9-*cis* retinoic acid; (**b**) expression of *STAR*, *CYP17A1* and *MC2R* after 24 h incubation with 10 nM ACTH and 10 nM 9-*cis* retinoic acid (RA); (**c**) expression of *RARA*, *RARB* and *SREBP1* after 24 h incubation with 10 nM ACTH and 10 nM 9-*cis* retinoic acid (RA). Data is expressed relative to ACTH-treated wells: equal to 100% for ACTH-stimulated cortisol secretion and equal to 1 for ACTH-stimulated gene expression; Grey bar: 10 nM ACTH, black bars: 9-*cis* retinoic acid (RA). *p < 0.05 vs ACTH.

9-*cis* Retinoic acid blunted ACTH-stimulated *MC2R* expression by roughly 50% (Fig. 2b) and did not affect the ACTH-induced cortisol response (Fig. 2a) nor ACTH-induced changes in *CYP17A* and *StAR* (Fig. 2b). This was replicated at analysis of gene expression normalized to control wells, as the changes in steroidogenic gene expression during retinoic acid-ACTH co-incubated wells were comparable to wells incubated with ACTH alone (Table 1). As regards modulation of retinoic acid receptors, expression of *RARA* and *RARB* expression was further enhanced by retinoic acid in ACTH-stimulated wells (Fig. 2c; Table 1). No significant changes compared to ACTH alone were observed for the genes involved in retinoic acid action (*NR1H3, PPARD, NR2F1, mt-ND1* and *mt-ND6*, Table 1).

We analysed cortisol secretion during ACTH/retinoid co-incubation by two approaches: the effect of 9-*cis* retinoic acid on the cortisol response to ACTH was compared to cortisol leves with 10 nM ACTH and the retinoid (10 nM: 92.2 ± 12.8% ACTH; 100 nM: 115.3 ± 16.8% ACTH; 1 µM: 123.6 ± 13.3% ACTH, all comparisons N.S., Fig. 2a) and to cortisol levels with each 9-*cis* retinoic acid concentration without ACTH (10 nM: 112.9 ± 13.7% RA; 100 nM: 144.6 ± 28.1% RA; 1 µM: 158.6 ± 32.5% RA, all comparisons N.S.); of note, the expected increase in cortisol levels with ACTH is over 300% of unchallenged wells (see above).

## Discussion

Retinoids are known modulators of adrenal embryonic development^1,16,17^ but effects appear to extend beyond adrenal organogenesis. All-*trans* and 9-*cis* retinoic acid have been shown to stimulate steroidogenesis in both adrenal and gonadal murine models^18–20^. Conversely, these retinoid agonists proved inhibitors of corticosteroid secretion and adrenal cell proliferation in mouse and human neoplastic adrenal cell lines^5,6^; on note, retinoic acid signalling pathway stands out in adrenal tumor microarray and miRNA analysis^21^. Retinoids act mainly via homo- or heterodimerization of ligand-activated RAR and RXR receptors with all-*trans* retinoic acid and 13-*cis* retinoic acid acting as RAR agonists and 9-*cis* retinoic acid binding both RAR and RXR^3^. These receptors may also heterodimerize with other nuclear receptors, such as PPAR gamma and LXR, or act—as has been shown for 9-cis retinoic acid- on mitochondrial RXRs^22^, thus adding an additional layer of complexity to retinoid action.

Retinoids has also been shown to affect tumoral corticotrope cells, corticotropes being the main regulators of adrenal cortisol secretion. In fact, studies on both the murine corticotrope cell line AtT-20 and in human adenomatous corticotrope primary cultures^5,8–10^ revealed that both all-*trans* and 9-*cis* retinoic acid can inhibit ACTH synthesis and secretion. Both bone morphogenic protein 4 (BMP4), a transcription factor involved in pituitary tumorigenesis, and COUP-TFI, a negative regulator of retinoic acid response pathways, modulated the action of all-*trans* and 9-*cis* retinoic acid action in tumoral corticotropes^5,8,23^. Interestingly, long term (48–192 h) incubation of AtT-20 cells with tambicarotene (Am80), a synthetic RAR alfa and beta agonist, revealed increased ACTH secretion as well as *Pomc* and *Tpit* expression^24^; at 24 h—the time frame evaluated in other experiments^5,8–10^- tambicarotene did not affect ACTH secretion and *Pomc* secretion, suggesting different involvement of RARs and RXR over time.

This evidence led to clinical trials with tretinoin and isotretinoin in patients bearing an ACTH-secreting pituitary adenoma, i.e., Cushing’s disease^11,12^, with promising results. So far, over 20 patients with this severe endocrine disorder have been tested and contaiment of cortisol excess could be observed in up to one third of patients, much like it occurs with other pituitary-acting drugs^13,14^. Interestingly, although the rationale for efficacy of retinoids in these patients rests on evidence collected on the tumoral corticotrope—thus inhibition of ACTH is expected to drive the reduction in adrenal secretion-, the decrease in cortisol levels appeared more pronounced and not strictly parallel to ACTH concentrations. This has been shown to occur with other drugs aimed at the pituitary but, given the known link between retinoids and the adrenal gland, could also be due to a direct action on adrenal cortex cells.

We therefore decided to pursue this avenue of investigation and tested 9-*cis* retinoic acid first in normal human adrenal tissue. Our study demonstrated that 9-*cis* retinoic acid stimulates spontaneous cortisol secretion as well as synthesis of *STAR*^15^, the rate limiting enzyme for adrenal cholesterol availability, in adrenal cells. At the same time, 9-*cis* retinoic acid markedly blunted expression of the ACTH receptor, i.e., melanocortin type 2 receptor MC2R, and reduced upregulation of *MC2R* induced by ACTH itself. The effects of 9-*cis* retinoic acid on adrenals appeared therefore two-fold: enhancement of spontaneous cortisol secretion and reduction of the ACTH receptor synthesis, suggesting a modulatory role in intraadrenal negative feedback regulation.

In patients with Cushing’s disease, adrenals are continuously exposed to elevated ACTH levels and it stands to reason that the effect of 9-*cis* retinoic acid on ACTH receptor expression may come to play a major role. Of note, long-standing exposure to excess ACTH usually leads to the development of hyperplastic adrenals in these patients^25^, attesting to the preeminent role of ACTH on adrenal secretion and trophism. Given the above, we decided to expand upon our previous findings and investigate the effect of 9-*cis* retinoic acid on adrenals from patients with Cushing’s disease.

Incubation with 9-*cis* retinoic acid led to doubling of spontaneous cortisol secretion in primary cultures established from adrenal tissues collected patients with Cushing’s disease, indicating that the stimulatory effect observed in normal adrenal tissue^15^ is mantained in hyperplastic adrenal cortex. Ancillary to increased cortisol secretion, we observed an increase in *STAR* expression—thereby enhancing cholesterol flux to the mitochondrion^26^- in keeping with results obtained with all-*trans* and 9-*cis* retinoic acid on gonadal and adrenal steroidogenic cell models^15,19^. 9-*cis* Retinoic acid also increased *CYP17A1* expression, thus potentiating microsomial steroidogenesis; similar results have been reported with all-*trans* retinoic acid in a murine tumoral Leydig cell line^27^. As regards the first enzyme of the steroidogenic cascade, cholesterol side-chain cleavage or P450SCC, a primary target of ACTH stimulation, no changes in *CYP11A1* expression or protein levels with either retinoid have been reported by us^15^ and other investigators^18,27^.

9-*cis* Retinoic acid also acted upon known targets such as its own receptors, *RARA* and *RARB*^28^, *DAX-1,* a transrepressor crucial to adrenal development^17^, and *SREBP-1*, a sterol regulatory element^29^. Of note, DAX-1 is a major inhibitor of *STAR*^30^ and both *STAR*^31^ and *CYP17A1*^32^ are modulated by SREBP-1. Altogether, this evidence suggests that 9-*cis* retinoic acid stimulates cortisol secretion via a concerted involvement of DAX-1, SREBP-1, STAR and CYP17A1 in hyperplastic adrenal cells.

In contrast, the most striking effect observed during 9-*cis* retinoic acid and ACTH co-incubation was the marked reduction in ligand-induced upregulation of the ACTH receptor^33–35^. The ACTH receptor is crucial to cortisol secretion, indeed, mutations in *MC2R* are associated with severe and often fatal cortisol deficiency^36^. In this context, our findings on *MC2R* in hyperplastic adrenal tissues collected from patients with Cushing’s disease confirm and extend results obtained during ACTH stimulation in normal adrenals^15,34,35^: in addition to *MC2R*, ACTH proved a strong inducer of *STAR*, *CYP17A1* and *LIPE*, the hormone-sensitive lipase crucial to adrenal steroidogenesis^37^.

The stimulatory effect of 9-*cis* retinoic acid on cortisol secretion and steroidogenic enzyme expression could not be observed in wells co-incubated with ACTH. In fact, cortisol secretion was comparable to levels observed with ACTH alone for 9-*cis* retinoic acid concentrations up to 1 μM as were *DAX-1*, *STAR* and *CYP17A1* expression. It is tempting to speculate that the reduction in *MC2R* induced by 9-*cis* retinoic acid dampens the adrenal response to ACTH, thus overriding its stimulatory effect on spontaneous cortisol secretion. In our previous study on normal adrenal tissue, the stimulatory effect of 9-*cis* retinoic acid on ACTH-stimulated cortisol secretion and *STAR* expression was modest or not significant^15^. It stands to reason that the stimulatory effect is abolished in ACTH-dependent adrenal hyperplasia, given the preeminent role of ACTH and, thus, MC2R.

Interestingly, ACTH also increased retinoic acid receptor beta (*RARB*) expression and, further, ligand-induced upregulation of both receptor isoforms was enhanced during ACTH/9-*cis* retinoic acid co-incubation. Of note, *RARB* has been shown to be more sensitive than *RARA* to upregulation by retinoids in some cell models^28^. Given that the adrenal gland itself produces endogenous retinoid acid^16^, these changes could come into play in an intraadrenal retinoic acid-ACTH circuit.

In patients with Cushing’s disease, cortisol hypersecretion is driven by ACTH produced by tumoral corticotropes. Although plasma ACTH concentrations are not always markedly elevated, they prove sufficient to determine excess cortisol secretion by the adrenal. In fact, the adrenal *MC2R* is believed to be upregulated in these patients by virtue of long-standing ACTH stimulation and, further, markedly reduced *MC2R* expression underlies the absent cortisol response to ACTH testing in patients with Cushing’s disease submitted to surgery^38^. Along the same line, the reduction of *MC2R* induced by 9-*cis* retinoic acid could play a major role in its therapeutic efficacy in Cushing’s disease. As mentioned above, the decrease in cortisol levels observed in patients treated with both tretinoin^11^ and isotretinoin^12^ appeared greater and not strictly time-related to changes in ACTH concentrations. The decrease in ACTH is expected by virtue of its action in human tumoral corticotropes^5,10^ whereas the observed down-regulation of *MC2R* could contribute to explain a greater decrease in cortisol.

In conclusion, our findings indicate that although 9-*cis* retinoic acid stimulates unchallenged cortisol secretion, in presence of ACTH the decrease in adrenal ACTH receptor overrides this effect. Thus, an adjunctive, adrenal action could play a role in the efficacy of retinoids in patients with Cushing’s disease.

## Material and methods

### Adrenal cultures

Adrenals obtained from 6 patients with Cushing’s disease submitted to adrenalectomy were established in culture according to our usual protocols^15,39^. In brief, the adrenal medulla was carefully removed and adrenal cortex fragments were minced, digested in 0.1% collagenase, plated at approx. 300,000 cells/well, incubated in DMEM supplemented with 10% fetal bovine serum and antibiotics for 3–5 days to allow attachment.

### Treatments and assays

Treatment were performed in DMEM containing 0.1% BSA. Cells were incubated with 10 nM, 100 nM and 1 μM 9-cis retinoic acid (Sigma Aldrich, St. Louis, USA) with or without 10 nM ACTH for 24 h. Pregnenolone (10 μM) was included in the test medium in order to promote steroidogenesis^40^. Both pregnenolone and retinoic acid were dissolved in 100% ethanol and diluted 1000-fold in DMEM; equal volumes of ethanol were added to control wells. Treatments were performed in triplicate for each adrenal specimen. Cortisol in medium was measured using Coat-A-Count radioimmunoassay (Siemens Healthcare Diagnostics, Erlangen, Germany) and normalized to unchallenged or ACTH-stimulated wells, respectively, given the considerable variability in cortisol medium concentrations among specimens (from 360 ng/ml to 990 ng/ml after 24 h incubation).

### Quantitative real-time PCR

RNA was extracted from plated cells using TRIzol reagent (Life Technologies, Carlsbad, USA) according to the manufacturer's instruction. The amount and quality of RNA were checked on nanophotometer (Implen GmbH, München, Germany) and 100 ng RNA reverse-transcribed with Superscript Vilo cDNA Synthesis Kit (Life Technologies, Carlsbad, USA). Quantitative Real-Time PCR was performed on a 7900 HT sequence Detection System (Applied Biosystems, Foster City, USA), using the Platinum Quantitative PCR Supermix-UDG with ROX (Life Technologies, Carlsbad, USA) and TaqMan assays. The following genes were evaluated: *STAR* Hs00264912_m1, *MC2R* Hs00300820_s1, *NR1H3* (LXRa) Hs00172885_m1, *NR0B1* (DAX-1) Hs00230864_m1, *PPARD* Hs00987011_m1, *NR2F1* (COUP-TF1) Hs00818842_m1, *CYP17A1* Hs01124136_m1, *LIPE* Hs00943410_m1, *RARA* Hs00940446_m1, *RARB* Hs00977140_m1, *mt-ND1* Hs02596873_s1, *mt-ND6* Hs02596879_g1, *NR5A1* (SF-1) Hs00610436_m1, *SREBP1* Hs01088679_g1 and normalized to *RPLP0* Hs99999902_m1. Expression data was analyzed as 2^−ΔΔCt^ and expressed as fold change vs control or ACTH. Changes in gene expression were evaluated in wells treated with 10 nM 9-*cis* retinoic acid.

### Statistical analyses

Data is expressed as mean ± standard error of the mean (S.E.M.) relative to unchallenged or ACTH-stimulated wells for each adrenal specimen. Comparisons were performed using Wilcoxon's signed-rank test and statistical significance accepted at p ≤ 0.05.

### Study approval

The study was approved by the Ethical Committee of the Istituto Auxologico Italiano (project #02C402) and carried out according to guidelines established by the Declaration of Helsinki.

### Informed consent

Informed consent for secondary use of surgical tissues obtained from patients by the referring physician prior to surgery.

## Data Availability

The datasets generated during the current study are available from the corresponding author on reasonable request.
